# Application of Nanoparticles and Melatonin for Cryopreservation of Gametes and Embryos

**DOI:** 10.3390/cimb44090276

**Published:** 2022-09-05

**Authors:** Hyun-Woo Choi, Hoon Jang

**Affiliations:** 1 Department of Animal Science, Jeonbuk National University, Jeonju 54896, Korea; 2 Department of Life Sciences, Jeonbuk National University, Jeonju 54896, Korea

**Keywords:** cryopreservation, cryoprotectant, melatonin, nanoparticles, improving fertility

## Abstract

Cryopreservation of gametes and embryos, a technique widely applied in human infertility clinics and to preserve desirable genetic traits of livestock, has been developed over 30 years as a component of the artificial insemination process. A number of researchers have conducted studies to reduce cell toxicity during cryopreservation using adjuvants leading to higher gamete and embryo survival rates. Melatonin and Nanoparticles are novel cryoprotectants and recent studies have investigated their properties such as regulating oxidative stresses, lipid peroxidation, and DNA fragmentation in order to protect gametes and embryos during vitrification. This review presented the current status of cryoprotectants and highlights the novel biomaterials such as melatonin and nanoparticles that may improve the survivability of gametes and embryos during this process.

## 1. Introduction

Assisted reproductive technologies (ARTs) have improved considerably over the past 40 years, including the use of cryopreservation in the field of reproductive biotechnology [1,2,3,4]. Cryopreservation is a method of preserving the structure and function of cells and reducing their metabolism and enzymatic activity while they are maintained at cryogenic temperatures [3]. Cryopreservation of human sperm has been well-established [5], whereas the ability to preserve oocytes remains elusive due to supply difficulties, as well as ethical and legal issues [6]. In particular, embryos are very difficult to cryopreserve because of their size, survival sensitivity, and multi-compositional structure [7,8]. Several studies have reported that aberrant effects such as genotoxicity, necrosis, and abnormal apoptotic activity are responsible for the cytotoxicity observed in gametes and embryos during cryopreservation and vitrification [9,10,11,12]. To overcome these impediments, a number of researchers have studied the effect of co-treatment of gametes and embryos with protective adjuvants and freezing media, which was found to reduce toxicity as well as increase the success rate of artificial insemination. As a result, several supplements have been developed to improve cryopreservation and other ARTs.

Several organisms that live in extremely cold temperatures, including bacteria, fungi, microalgae, insects, crustaceans, and fish, produce specific proteins, called antifreeze proteins (AFPs) or ice-binding proteins (IBPs), that can inhibit ice recrystallization [9,11,13,14,15,16,17,18,19]. In a subzero temperature environment, these proteins interact with ice crystals and regulate thermal hysteresis in order to stabilize the cell membrane [20]. These proteins are essential to the protection of cellular membranes from freezing. A number of researchers have sought to use these proteins, and have searched for additional cryoprotectants, to reduce cellular damage due to freezing during cryopreservation [19,21].

Melatonin (N-acetyl-5-methoxytryptamine) is a hormone secreted from vertebrate pineal gland and regulates homeostasis of reproductive tissues such as ovary and placenta [22] through receptor-mediated signaling pathways [23]. Recent studies have reported that melatonin plays a critical role in the cryopreservation of sperm [24,25,26,27,28,29,30,31,32,33,34,35,36,37,38,39], oocytes [40,41,42,43,44,45,46], and embryos [47,48,49,50,51] in various species. In addition, melatonin is an antioxidant that acts as a free radical scavenger and affects G-protein-coupled receptor-related signaling transduction [23,52]. A number of researchers have identified that melatonin is a key contributor to the maintenance, proliferation, differentiation, and survival of germ cells, as well as somatic cells [53,54,55,56]. Melatonin has also displayed an antioxidant effect on chemotherapy-induced ovarian follicle dysfunction in mice ovaries [57,58,59]. Because cells are exposed to extreme oxidative stress during cryopreservation, the positive antioxidant effect of melatonin on cryopreservation is being studied. Consequently, numerous studies have been performed to examine the beneficial effect of various antioxidants on cryopreservation.

Nanoparticles (NPs) have recently been applied in the fields of bioscience and biomedicine [60,61,62]. Nanotechnology, which utilizes cellular response modulating, has been an excellent impactful method in numerous aspects of biology, including artificial insemination (AI) in the livestock industry [63]. NPs are currently being studied for their potential use as cryoprotectants, and a cryoprotective effect has already been reported in several animals [64,65,66,67,68]. The potential positive impacts of nanotechnology have led to further research into its use in cryoprotection, and with the development of nanotechnology, it is necessary to find a way to use it continuously.

A number of cryopreservation techniques have been tested, such as the slow freeze-thaw and rapid freeze-thaw of gametes and embryos [69,70]. In addition, cryoprotectants divided into permeable agents and non-permeable agents according to their ability to migrate to the cell membrane are continuously being developed [71]. Researchers have studied the mechanisms of several cryoprotectants and have found that some are toxic when applied to gametes [72,73,74]. Thus, there is growing interest in reducing the toxic effect of germ cells during the freezing-thawing procedures using cell-specific cryoprotectants, in order to increase the effectiveness of gamete and embryo cryopreservation. The following review presents an updated summary of the approaches used to reduce cytotoxicity during the cryopreservation of gametes and embryos.

## 2. Technical Problems and Solutions of Cryopreservation of Gametes and Embryos

Following cryopreservation, cell viability mainly depends on the level of intracellular ice crystallization that occurs during the freezing process. Cryopreservation methods are divided according to freezing speed, including slow freeze (~0.5 °C/min), rapid freeze (~400 °C/min), ultra-rapid freeze (~2500 °C/min), and vitrification (~20,000 °C/min) [75,76]. When freezing an extender between −1 and −15 °C, crystallized ice forms around cells while the inside of cells does not freeze. After the water is transferred to the extracellular extender, the cells become dehydrated due to hyperosmotic pressure [76]. As this process results in cellular damage and toxicity, optimal cryopreservation extenders, such as additive agents, have been developed. Permeable additive agents including Dimethyl sulfoxide (DMSO), glycerol, 1,2-propanediol, and ethylene glycol (EG) have been used to cryopreserve gametes due to their lipophilic properties, as well as their ability to easily transfer into cellular membranes, control an osmotic gradient, and inhibit the formation of ice [77]. However, these agents cause lipid peroxidation (LPO) which has an adverse effect on sperm during the thawing process [78]. Increased LPO results in significant physicochemical damage to cellular organelles and to the membrane of sperm, causing low sperm motility, reduced mitochondrial activity, and sperm toxicity [78]. Consequently, non-permeable cryoprotectants such as carbohydrates (glucose, trehalose, and sucrose) have been examined. The high molecular weight of carbohydrates prevents their transfer into the cell membrane, which facilitates high-speed freezing. Carbohydrate-based cryoprotectants may promote cellular dehydration, avoid ice crystallization, and increase membrane stabilization [79]. Nevertheless, there are additional impediments to overcome, such as cell toxicity induced by reactive oxygen species (ROS), as well as DNA alteration, cytoskeletal dysfunction, and mitochondrial damage that occurs during gamete cryopreservation [80,81,82]. Various techniques and protective substances must be developed to increase the effectiveness of cryopreservation of reproductive cells.

As aforementioned, cryopreservation is used to preserve various cells at low temperatures, and the process negatively affects the structure, function, and metabolic activity of cells. Therefore, many researchers have sought to determine the optimal conditions for cryoprotectants to prevent physical cell damage caused by osmotic stress or chemical cell damage such as membrane receptor-related signaling transduction [79]. Vitrification occurs when a highly concentrated solution is frozen rapidly without changing its state, such as the crystallization of a liquid into a solid-like substance without the formation of ice crystals in the cytoplasm, and usually requires high concentrations of cryoprotectant to reduce the risk of ice crystallization [83,84].

There are two types of vitrification protocols: open and closed. The open protocol occurs when cells are completely submerged in liquid nitrogen using a low-volume glass capillary tube, pulled straws, cooper devices, and loops. A number of trials have been conducted using the open vitrification protocol to ensure that in vitro fertilization (IVF) using cryopreserved oocytes is as effective as IVF using fresh oocytes [85,86]. Despite the similar success rates in open IVF using frozen oocytes and fresh oocytes, asepsis-related problems have been observed due to cross-contamination between vitrificated oocyte samples and microorganisms in liquid nitrogen [87,88,89]. To solve this issue and minimize surrounding contaminants, liquid nitrogen was sterilized using UV light, or additional pretreatment was applied [89,90]. However, due to the difficulty of implementing a perfectly aseptic system, in-straw vitrification was developed. The in-straw vitrification can be stored in liquid nitrogen in a hermetically sealed state and is particularly useful for aseptic frozen storage of sperm for artificial insemination.

Vitrification, currently the fastest freezing method which was introduced in 2007, reduces damage to cell structure and improves cell viability. This method is performed at a high freezing rate and causes rapid dehydration using a high osmotic extender. Fast freezing occurs when the cells are at subzero temperatures, which is called the osmotic balance stage. During this process, the viscosity of the extender increases until the cytoplasm changes from a liquid to a solid, and all water is displaced from the cells without the intracellular formation of ice crystals. Accordingly, a cryopreservation technique was developed using cryoprotectant proteins such as antifreeze proteins. However, cryoprotection studies that used sperm initially failed due to osmotic damage and toxicity of the highly permeable cryoprotectants [84]. Zachariassen et al. reported that most highly permeable cryoprotectants induce toxicity and osmotic damage due to sperms’ size down and low levels of cytoplasm [91]. The first study that used a cryoprotectant protein to improve sperm cryopreservation showed that antifreeze glycoprotein type 1 enhanced the survival rate and motility of ram sperm [92]. These reports suggest that the concentration of cryoprotectants, freezing speed, and optimized device systems such as straws or tubes are critical aspects of the cryopreservation process.

Methods of cryopreserving sperm are divided into slow freezing and rapid freezing. Rapid freezing requires much less time than slow freezing and improves sperm motility and survival rates after cryopreservation [70]. Cryopreservation, which disturbs the intracellular environment, may result in decreased sperm motility and fertility due to damage to the acrosome and cytoskeletal changes [70]. It has been reported that α-tubulin plays an important role in sperm motility through acetylation as a major cytoskeleton constituting the tail of sperm [93], and the levels of the α-tubulin are abnormally elevated in cryopreserved samples [94]. In contrast, Wang et al. reported that the expression of cytoskeletal proteins such as mitochondrial aconitate hydratase, Tektin-1, and vimentin was significantly decreased in cryopreserved human sperm [95]. The tektin protein family is a filamentous protein that is mainly present in the tail of sperm and has been reported as a cytoskeleton that plays an important role in sperm motility [96], and Vimentin is a fibrous protein distributed in the inner membrane that protects sperm morphology from physical shock caused by sperm activity [97]. Vitrification dramatically enhances sperm activity compared to the slow freezing method. It has been reported that when vitrification is implemented, sperm motility and the conserved membrane potential of mitochondria are significantly improved compared to slow freezing [98]. It has been reported that freezing sperm via vitrification results in significantly improved membrane integrity and less DNA fragmentation compared to slow freezing [99,100].

Methods of cryopreserving oocytes can be mainly divided into slow-cryopreservation and vitrification. The slow-cryopreservation process freezes oocytes at a slow rate to prevent rapid heat shock that contributes to cell damage and requires a relatively low concentration of cryoprotectant [101,102]. Vitrification is the rapid crystallization of a liquid into a solid-like substance without the formation of ice crystals in the cytoplasm. Like sperm, DNA damage associated with ROS production in oocytes during the freeze-thaw process induces apoptosis and reduces oocyte viability. Therefore, the development of cryoprotectants to be used in the vitrification process are essential for the efficient cryopreservation of gametes.

During cryopreservation, embryos are subjected to significant stress via osmotic pressure, extreme temperature change, and oxidative stress, as well as physical damage caused by ice crystallization. To prevent this, a number of researchers are trying to develop optimal cryopreservation techniques using various types of cryoprotectants such as several types of AFPs. The various aforementioned agents are commonly used for the cryopreservation of embryos as well as gametes. However, the cell permeability and cytotoxicity of embryos are different than single-celled gametes, requiring varied concentrations of treatment conditions and cryoprotectants. For example, it has been reported that in certain species, cryoprotectant treatment only reduces cytotoxicity at lower temperatures, in high concentrations, or in a time-dependent manner [103]. In particular, if the cryoprotectant demonstrates effective permeability, it should be used at the initial stage of cryopreservation, and if it shows weak permeability, it should be used at a later stage.

## 3. Biomaterials in Gamete and Embryo Cryopreservation

The cryopreservation of human oocytes and follicles was first reported in 1986 [104] though the survival rate of the oocyte was low due to technical deficiencies [105]. Oocytes are difficult to cryopreserve due to their high sensitivity to cytoplasmic function, low resistance to ice crystal formation, and small surface area to volume ratio [106]. Several reports have indicated that additional obstacles to the cryopreservation of oocytes include their physical properties such as permeability and microtubule stability [107,108,109,110], as well as structural features such as their zona pellucida [111,112]. A pair of 1994 studies reported that oocytes have the ability to maintain their morphology and functional integrity during cryopreservation [113,114]. However, effective cryopreservation of oocytes still requires the development of additional reliable techniques and protective adjuvants.

The cryopreservation of embryos was successfully performed in 1972 using morula stage mice embryos [115], and the transplantation of cryopreserved embryos into humans was achieved in 1983 [116]. With the recent advances in implantation technology using frozen embryos, it has been possible to produce genetically superior livestock or to overcome infertility through artificial insemination by transferring embryos [75,83,117,118,119,120,121]. In addition, infertility in young female cancer patients may be prevented by cryopreserving embryos prior to chemotherapeutic treatment [122].

The first cryopreservation of human sperm was successfully performed in 1949 using glycerol at −74 °C [123]. Sperm have limited cellular functions compared to normal cells, and their unique tail makes it difficult for their structure to recover after cryopreservation [5,124]. Oxidative stresses also affect the lipid composition of the cytoplasmic membrane, intracellular proteins, and extremely condensed genomic DNA of sperm, which decrease the motility, viability, and fertility of sperm [5,124]. During cryopreservation, sperm produce ROS and exhibit significantly increased lipid peroxidation, membrane damage, and DNA fragmentation due to the reduced level of antioxidant activity [5,124,125]. In particular, after the freeze-thawing process has been completed, DNA damage associated with ROS production leads to apoptosis and decreases sperm viability [126]. Researchers have utilized protective adjuvants and implemented optimal temperatures and periods of time to be used in the freeze-thawing stage to inhibit the production of ROS [26,84,92,127,128,129,130,131]. The optimized protocols [132,133], as well as antioxidants including L-carnitine [134], cysteine [135], vitamin E-related NPs [136], and melatonin [25,125] have been reported to scavenge ROS during sperm cryopreservation. Nonetheless, more research needs to be done regarding the potential benefits of antioxidant treatment.

### 3.1. Melatonin

Considering that increased ROS production during cryopreservation is responsible for the deterioration of gamete quality after thawing, various antioxidants have been examined for the cryopreservation of gametes from various animals [136]. Melatonin, a type of indoleamine and free radical scavenger, has been reported to exhibit various antioxidative effects through G-protein-coupled receptors (GPCR). Recently reported studies have focused on the protective effect of melatonin in maintaining fertility in several species of animals and humans [25,27,28,29,30,31,34,39,137,138,139]. Even in humans, melatonin has been reported to be very useful as a sperm cryoprotectant and it has a significant effect on the toxicity and damage recovery of frozen sperm. Three receptors for melatonin (MT1, MT2, MT3) have been identified, and most mammalian cells have MT1 and MT2 [23]. In germ cells and related somatic cells, it has been reported that MT1 is present in the cell membrane [140]. Our previous study also discovered that both MT1 and MT2 were observed in mouse primordial follicles and oocytes [57,58]. Receptors in these cells mediate the effects of melatonin. Espino et al. reported that melatonin has a protective effect on human sperm activity via regulating caspase-3 and ERK signaling [139]. Interestingly, several studies have shown that melatonin had a crucial role in the protection of germ cells via the signaling pathway of non-receptor mediation [125,141]. Based on these findings, it is possible that the effects of non-receptor-mediated melatonin may impact ROS scavenging, enzymatic antioxidant activity, and molecular conservation against oxidative stress. As aforementioned, since cryopreservation causes oxidative stress and various intracellular damage, as well as generates ROS, melatonin may play an important role in cryopreservation. A study that analyzed the mechanism of melatonin showed that melatonin significantly enhanced the expression of Nox5 and Nrf2, as well as increased the ratio of Bcl2/bax [23,52] in neuroendocrine regulation.

Research on the sperm cryopreservation of melatonin has been actively conducted for about a decade. In 2011, Succu et al. reported that the addition of melatonin to the freezing extender enhanced intracellular ATP concentration and DNA integrity in ram sperm [26]. They also found that cryopreserved sperm with melatonin induced a faster rate of division in embryos constructed after IVF compared to controls. Since then, researchers have conducted numerous studies on the effects of melatonin on freezing human sperm. Karimfar et al. reported that melatonin significantly decreased the production of ROS and malondialdehyde in cryopreserved human sperm [27], and Deng et al. presented that melatonin promotes the expression of heat shock protein 90 via MT1 signaling pathway in human sperm [32]. Najafi et al. suggested that melatonin might exert its effects through the PI3K-AKT signaling pathway in human sperm [33], Pariz et al. discovered that the combination of melatonin and caffeine has a dramatically protective effect on human sperm cryopreservation [28]. Various animal studies have also demonstrated the cryoprotective effect of melatonin. In the bull, melatonin improves the success rate of IVF by enhancing cryopreserved sperm quality [29,35,37]. In the ram, melatonin inhibits the mitochondrial permeability transition pore opening by MT1-related PI3K/AKT/GSK3b signal activation [34]. Additionally, melatonin and Myo-inositol mixture have a critical effect on sperm activation during cryopreservation in goat sperm [39]. In pigs, a melatonin and butylhydroxytoluene mixture successfully reduced reactive nitrogen species and oxidative stress during a sperm vitrification procedure [36]. In rats, the melatonin-selenium combination increased sperm motility and mitochondrial activity after cryopreservation [38]. In rabbits, melatonin significantly induced glutathione peroxidase, superoxide dismutase, and catalase in sperm after cryopreservation [25].

As mentioned earlier, studies on the cryopreservation of oocytes by melatonin have been conducted only recently because it is difficult to collect samples compared to sperm. In particular, studies using mouse animal models have been mainly conducted compared to humans or large animals. Zhang et al. reported that the supplementation of melatonin increased the development of vitrified mouse MII oocytes by reducing the transition from maternal to zygotic and scavenging ROS [40]. Additionally, one study reported that resveratrol and melatonin significantly improved in vitro maturation of cryopreserved germinal vesicle (GV) oocytes in mice [41]. Doroudi et al. reported that melatonin and human follicular fluid mixture enhanced the cleavage rate of the two-cell stage of the embryo which was fertilized using vitrified mouse oocytes [43]. Furthermore, Pan et al. identified that melatonin promotes the first cleavage of parthenogenetic zygotes via increasing the mitochondrial membrane potential of vitrified mouse oocytes [44]. In pigs, Tang et al. reported that a melatonin-glycine mixture improves the development of vitrified GV oocytes via regulating osmotic stresses [45]. In 2021, Zhang et al. presented that melatonin maintains the permeability of the oolemma and suppresses oxidative stress through upregulation of aquaporin-1 expression in human vitrified oocytes [46]

In embryo cryopreservation, the embryo is very sensitive to oxidative stress because of its multicellular structure. Succu et al. reported that melatonin has beneficial effects on embryo viability and hatching rate by regulating ATP concentration in the ram [48]. Mehaisen et al. also presented that melatonin successfully protected the morula stage of the vitrified embryo through the reduction of LPO and nitric oxide in the rabbit [49]. Additional studies discovered that melatonin enhanced the development of in vitro propagated early stages of mouse [50] and bovine embryos [51]. Studies on the effects of melatonin on the freezing of human embryos have not been reported due to ethical concerns. Taken together, it is suggested that melatonin as a cryoprotectant could successfully protect gametes and embryos during cryopreservation. Representative research using melatonin in various species, and cell types, are shown in Table 1.

### 3.2. Nanoparticles

NPs are molecules with a diameter of less than 100 nm which can be useful for protecting reproductive cells during cryopreservation due to their physical and chemical properties. Although the activity of NPs is limited by their size, charge, and hydrophobicity, their application as cryoprotectants may be beneficial in a variety of cells that have been subjected to severe stress during a freeze-thaw cycle. Continuous developments in the field of nanotechnology have enabled NPs to perform more specialized functions, such as providing antioxidant effects in the cryopreservation of human somatic cells [142]. NPs have shown the potential to ensure enhanced integration of various factors in cellular processes and physiological activity without interfering with normal biological processes. Various novel types of NPs have been used as adjuvants with promising biological properties, and safe characteristics, and appear to have facilitated high levels of physiological activity.

During the freezing-thawing cycle, increasing ROS production reduces cell metabolism and causes an acrosome reaction in sperm. A recent study reported that nano-micelles improved cryopreserved sperm quality and acrosome protein integrity in the human [143]. In addition, studies on the cryopreservation effect of sperm using various types of NPs have been conducted in several animal models. Nano-zinc oxide successfully decreased ROS levels and improved sperm survivability in the ram [144], and zinc-related NPs and selenium enhanced the ultrastructure of camel sperm during cryopreservation [66]. Selenium NPs also improved sperm quality and reduced apoptosis-related damage in thawed bull sperm [145]. Magnetic-conjugated NPs have been shown to successfully select superior sperm among metal-coated boar semen [146]. In addition, gold NP may have enhanced acrosomal integrity and reduced oxidative stress by scavenging ROS in the goat [64]. No toxic effect of NPs on sperm has been reported, but gold NPs have been shown to accumulate in the nucleus of sperm [64]. Recently, Rubio et al. showed that Vitamin E NPs suppressed oxidative stress and preserved mitochondrial activity by reducing ROS and LPO in cryopreserved deer sperm [136]. In addition, herbal-extracted NPs protect sperm during cryopreservation via their antioxidant effects. Extenders containing curcumin enhanced vitrified sperm quality in the rabbit [68] and bull [147]. In addition, studies on the protective effect of herbal NPs such as *Moringa oleifera*, *Alnus incana, Albizia harveyi*, and *echinacea* in sperm cryopreservation were reported in buffalo [148], ram [131,149], bull [150] and had a protecting effect in sperm cryopreservation.

The studies of oocyte and embryo cryopreservation have been relatively few reported compared to spermatozoa. In oocytes, Sheng et al. reported that hydroxyapatite nanoparticles can reduce the number of ice crystals during vitrification by reducing mechanical damage in porcine oocytes [151]. In addition, Abbasi et al. reported that the Fe_3_O_4_ nanoparticle modulated pluripotent gene expression during embryo development which was produced by vitrified oocytes in the mouse [152]. Based on the study of sperm, it is expected that the cryoprotective effect of NPs in oocytes and embryos will continue to be reported.

Overall, NPs which demonstrate protective effects have the potential to play a significant role in the survivability and activity of animal gametes during cryopreservation. Representative research using various NPs during cryopreservation of gametes in several species is shown in Table 2.

### 3.3. Previously Used Agents

Even before the cryopreservation study of melatonin and NP, adjuvant studies were conducted to increase the viability of cells during cryopreservation. Preliminary research examined antifreeze protein (AFP) that helps organisms survive in sub-zero temperatures [153,154]. This protein lowers the freezing point of the water surrounding the cell membrane to below the melting point, allowing organisms to survive in low temperatures [155]. AFPs interact with ice surfaces and suppress ice crystallization until the freezing temperature. Many studies have reported that AFP has a significant effect on the survivability of oocytes, sperm, and several tissues, including embryos, during vitrification [156,157]. In addition, several studies reported that there are diverse types of AFPs and that each AFP has a specific protective effect when exposed to different kinds of cells [158,159,160]. Vitrification without cryoprotectants changes the organization of the endoplasmic reticulum in oocytes, leading to a lack of cortical clusters that normally appear after oocyte maturation [161]. As both slow freezing and vitrification may decrease the number of cortical granules and alter mitochondrial morphology, various protectants such as DMSO, EG, and AFPs have been examined. Vitrification of oocytes using DMSO and EG has been shown to vary the distribution of inositol 1,4,5-triphosphate receptors when pig oocytes were thawed, and ultimately restored Ca^2+^ homeostasis [162]. Several studies have recommended that AFPs may be valuable candidates in cryopreservation to protect embryos from various injuries, due to their ability to associate with cellular membranes and their capacity to prevent ice crystallization [18,163]. During cryopreservation of bovine embryos, a type III AFP improved embryo survival and development in the morula stage [164], and extenders (including AFP) as well as technically modified prewarming significantly enhanced the viability of embryos [165]. In addition, the AFGP8-contained extender has been reported to have a protective effect against low temperature-mediated injury of bovine blastocysts [166]. In mouse embryo cryopreservation, AFP preserved embryos morphologically better than controls using only traditional cryoprotectants such as DMSO but did not confirm any effect on survival and subsequent development. However, Shaw et al. reported that cryopreserved four-cell embryos that contained type 1 or type 3 antifreeze proteins did not improve development and viability [118]. It has been reported that AFPs affect different animals in distinct ways. In rabbits, type 3 AFPs improved cryopreserved embryo survivability [167], but type 3 AFPs demonstrated no improvement in embryo cryopreservation in porcine embryos [159]. AFP also had no effect on the cryopreservation of equine embryos [119]. Therefore, continuous research on the establishment of novel cryoprotectants such as melatonin or NP is essential.

## 4. Clinical Approaches in Cryopreservation

From a clinical approach, physicians need to understand the details of cryopreservation in order to advise their patients regarding ART. However, clinical guidance and the patient consultation process are currently deficient, specifically related to the issues of how samples are gathered and whether or not cryopreserved sperm should be used. The number of samples to be cryopreserved depends on the quality of the provided sperm, which may be affected by the patient’s overall health. A recent study reported that among cancer patients, motility was the most affected attribute of sperm. Sperm motility in prostate cancer patients, in particular, has been found to be sufficient, whereas lymphocytic leukemia patients exhibited the lowest levels of motility of the groups that were studied [168,169]. The pathophysiologic study of sperm, before and after cryopreservation, may suggest specialized procedures for different types of cancer, as well as the need to use particular cryoprotection techniques for patients with certain cancers. In oocytes, cryopreservation has been primarily used for cancer patients who will be exposed to chemotherapy or radiation therapy. The preservation of embryos has become an aspect of treating young female cancer patients, as they may be at risk of post-treatment infertility. According to one study, more than 60% of the young cancer patients surveyed were concerned about infertility at the time of their diagnosis, and more than half wanted to have children after treatment [170]. Ovarian failure or menopause after cancer treatment is dependent on the dosage of neoplastic drugs, the age of the patient, and the amount of radiation exposure. For these reasons, it is of utmost importance for doctors to discuss infertility risks with cancer patients and review the different options for preserving fertility before starting treatment. One of the many options for preserving fertility is the cryopreservation of oocytes. Studying how to protect oocytes before and after cryopreservation may lead to preserving the fertility of female cancer patients.

## 5. Conclusions

**Figure 1:**
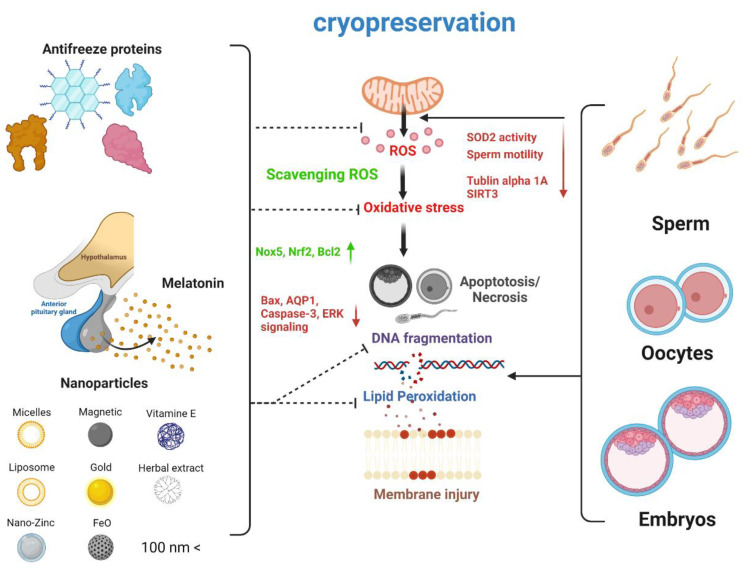
Main consequences of using cryoprotectants during cryopreservation. During cryopreservation, gametes and embryos are subjected to oxidative stress and physiochemical damage such as membrane injuries and DNA fragmentation due to rapid environmental changes. During cryopreservation, the number of reactive oxygen species (ROS) dramatically increases due to repressed SIRT3 and superoxide dismutase 2 (SOD2) activity. In particular, sperm motility is considerably reduced due to cytoskeletal alterations, caused by reduced levels of tubulin alpha 1A. Cryoprotectants can successfully reduce the number of ROS via scavenging, which is induced by antioxidant-relevant proteins such as Nox5, Nrf2, and Bcl2. In addition, cryoprotectants can reduce the expression of cell death-related proteins such as Bax, AQP1, and Caspase-3. Altogether, these various effects increase the efficiency of gamete and embryo cryopreservation by inhibiting lipid peroxidation and DNA fragmentation as well as limiting the production of ROS. Figure 1 summarizes the various effects of cryoprotectants. The figure is created with BioRender.com (accessed on 20 May 2022).

Various cryopreservation methods as well as gamete and embryos cryoprotectants have been developed to preserve fertility or superior traits of livestock. Many studies have reported improved viability and activity in gametes and embryos using various cryoprotectants. AFP is a representative cryoprotectant that has been widely used since the cell-freezing method was developed and enhances the survival of gamete and embryos after freezing. Although the effect of AFP varies widely depending on the cell type and biological species, as well as the fact that its protective mechanism has not been elucidated in most cases, AFP clearly plays an important role as a cryoprotectant. As substances that reduce the formation of physical ice crystals, cryoprotectants are utilized for the efficient removal of ROS, which rapidly increase during freezing and thawing. Melatonin, which has a remarkable ROS-removing effect by efficiently reducing oxidative stress, is a representative candidate. A number of studies have reported the inhibitory effect of melatonin on apoptosis, as well as its mechanism. It has been reported that antioxidative effects may also be successfully applied in gamete and embryo freezing. In addition, new types of cryoprotectants are being researched due to advances in nanoscience and nanotechnology. It has been reported that various types of NPs derived from natural products have a successful cryoprotective effect through various protective mechanisms as well as ROS removal. Lastly, as improved cryoprotectants are being actively developed by synthesizing artificial NPs, it is expected that a new approach to cell freezing and the development of innovative nano-bio technologies will be successful, and the efficiency of cryopreservation will be improved (Figure 1).

## Figures and Tables

**Table 1:** Research using melatonin during cryopreservation.

Type of Sample	Species	Ref.
Sperm	Human	[26,34]
Sperm	Bull	[26,34]
Sperm	Ram	[26,34]
Sperm	Goat	[39]
Sperm	Pig	[36]
Sperm	Rabbit	[25]
Sperm	Rat	[38]
Oocyte	Human	[46]
Oocyte	Horse	[42]
Oocyte	Mouse	[42]
Embryo	Cow	[51]
Embryo	Ram	[48]
Embryo	Rabbit	[49]
Embryo	Mouse	[50]

**Table 2:** Research using nanoparticles during cryopreservation of gamete.

Type of NPs	Type of Sample	Species	Ref.
Nano-micelles	Sperm	Human	[143]
Zinc Oxide	Sperm	Ram	[144]
Zinc Oxide and Selenium	Sperm	Camel	[66]
Selenium	Sperm	Bull	[145]
Iron oxide (Fe_3_O_4_)	Sperm	Boar	[146]
Gold-nanoparticle	Sperm	Goat	[63]
Vitamin E	Sperm	Deer	[134]
Curcumin	Sperm	Rabbit	[150]
Curcumin	Sperm	Bull	[151]
*Moringa oleifera*	Sperm	Buffalo	[152]
*Alnus incana*	Sperm	Ram	[153]
*Albizia harveyi*	Sperm	Bull	[154]
*echinacea*	Sperm	Ram	[130]
Hydroxyapatite	Oocyte	Pig	[155]
Iron oxide (Fe_3_O_4_)	Oocyte	Mouse	[156]

## Data Availability

Not applicable.
